# Comprehensive Review of Microbial, Plant, Biochar, Mineral, and Nanomaterial Solutions for Lead-Contaminated Wastewater

**DOI:** 10.3390/toxics13121082

**Published:** 2025-12-16

**Authors:** Aminur Rahman, Md Azizul Haque, Md Mahbubur Rahman, Pottathil Shinu, Muhammad Muhitur Rahman, Aftab Ahmad Khan, Sayeed Rushd

**Affiliations:** 1 Department of Biomedical Sciences, College of Clinical Pharmacy, King Faisal University, Al-Ahsa 31982, Saudi Arabia; spottathail@kfu.edu.sa; 2 Department of Biochemistry and Molecular Biology, Hajee Mohammad Danesh Science & Technology University, Dinajpur 5200, Bangladesh; helalbmb2016@hstu.ac.bd; 3 Department of Mechanical Engineering, Khulna University of Engineering & Technology, Khulna 9203, Bangladesh; mahbub_rahman@me.kuet.ac.bd; 4 Department of Civil and Environmental Engineering, College of Engineering, King Faisal University, Al-Ahsa 31982, Saudi Arabia; mrahman@kfu.edu.sa (M.M.R.); aabkhan@kfu.edu.sa (A.A.K.); 5 Department of Chemical Engineering, College of Engineering, King Faisal University, Al-Ahsa 31982, Saudi Arabia; mrushd@kfu.edu.sa

**Keywords:** lead toxicity, wastewater treatment, nanotechnology, heavy metal pollution, comparative analysis, eco-friendly solutions

## Abstract

Lead (Pb) pollution in wastewater is an immense problem for public health and the environment because it persists in the water bodies for a long period of time. Over the past years, many different techniques of Pb remediation have been discovered to eliminate Pb pollution. This systematic review analyzed the major findings of Pb removal from wastewater using microbial biosorption, agro-waste- and fruit peel-based adsorbents, plant-assisted phytoremediation, engineered biochars, clay and natural minerals, and nanomaterials. Each of these methods is critically reviewed in terms of removal efficiency, limitations, cost-effectiveness, how it works, how well it eliminates the problem, environmental compatibility, regeneration potential, and scalability, as supported by recent experimental and case studies. This review provides a comprehensive comparison of all the remediation methods in one framework. It also shows the potential of the integrated and hybrid systems, a combination of biological and high-technology material-based strategies, to reach high-performance Pb remediation in the long run. Therefore, the study aims to assist policymakers, environmental engineers, and researchers who are interested in finding a sustainable solution to Pb contamination by providing a comparative overview of the existing and recently developed remediation methods.

## 1. Introduction

**Figure 1:**
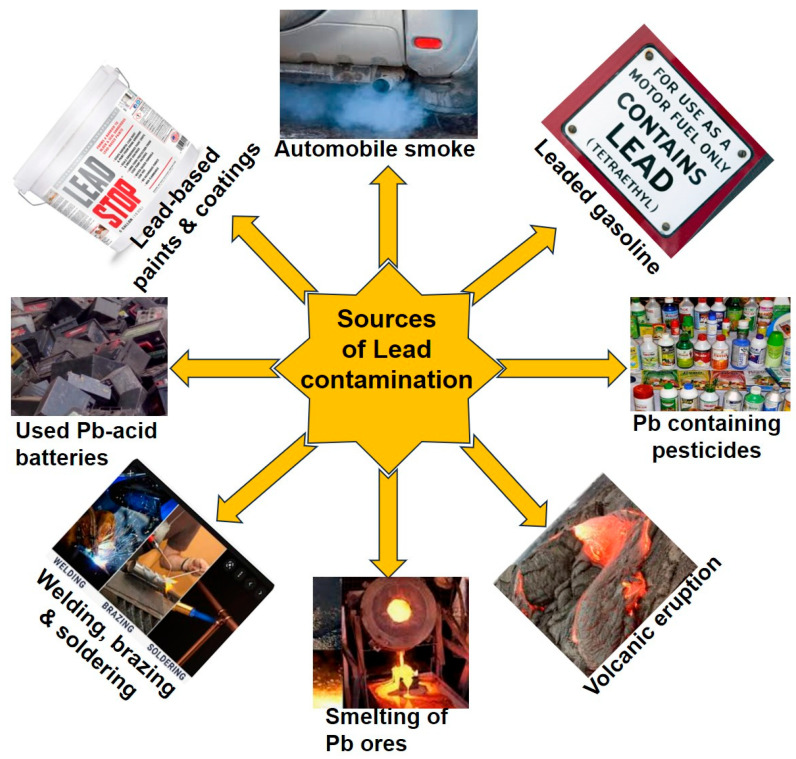
Sources of Pb contamination. The significant environmental sources of Pb pollution include automobile emissions, usage of leaded gasoline, pesticides containing Pb compounds, natural occurrences like volcanic eruptions, industrial activities, smelting of Pb ores, welding, brazing, soldering, improper disposal or recycling of used Pb-acid batteries, and the corrosion of Pb-based paints and surface coatings.

Water quality is under constant threat worldwide due to the discharge of heavy metals into water from industrial waste [1,2]. The rapid development of urbanization, industrialization, and agricultural intensification in the last century has greatly increased the release of heavy metals in the environment. Among these metals, lead (Pb) is the most widely used and also the most toxic metal [3,4]. Pb is a highly polluting element of aquatic ecosystems because Pb is non-biodegradable. The sources of Pb contamination include effluents from industries such as mining, battery manufacturing, metal smelting, ceramics, pesticides and fertilizers, volcanic eruptions, smelting of Pb ores, pigment production, petroleum refining, Pb-based paint, and other factories that use Pb [5,6,7,8] (Figure 1). Moreover, Pb can be found in many other products, including candies, cigarettes, wrappers, toys, and certain ethnic foods, such as dried grasshoppers. Some folk medicines, such as Azarcon, Bala, Greta, Golf, Ghasard, and Kandu, are sources of Pb. In addition, some cosmetics, such as Kohl (alcohol) and Surma, may contain Pb [9,10,11]. Also, some people exposed to Pb at work may bring it home on their clothes, shoes, skin, or hair [12].

**Figure 2:**
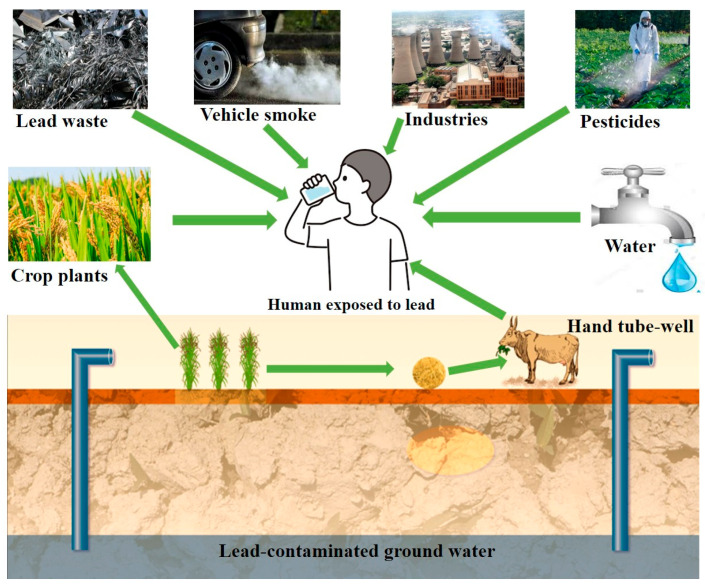
Diagram of Pb pollution in the ecosystem and the pathways humans are exposed to it. A schematic diagram of the diffusion of Pb contamination within the environment and the eventual end-product on human health. The arrows denote how humans are exposed to lead.

Pb can be ingested through vegetables and fruits grown in soils contaminated with high levels of Pb [13]. Additionally, meat and milk can be contaminated with Pb by supplying the Pb-contaminated animal fodder to cattle. By consuming this contaminated milk and meat, humans can ingest the toxic Pb pollutant. A schematic diagram of human exposure to Pb is demonstrated in Figure 2.

### 1.1. Toxicological and Environmental Effects and Regulations

Improper disposition and treatment of Pb-based wastes have contributed to the accumulation of Pb in water bodies and created critical environmental and community health hazards [14]. Pb pollution, in environmental matrices mostly in agricultural soils, water bodies, and air, is a serious global problem due to its high toxicity, non-biodegradability, and persistence. Pb accumulation in soils decreases the microbial biomass and enzymatic activity and changes soil physicochemical properties [1]. In aquatic environments, Pb leaching from industrial effluents and landfills contributes to contamination of the surface and groundwater. Even small amounts of Pb^2+^ ions may be accumulated in the flora and fauna of the aquatic environment, and this promotes biomagnification and bioaccumulation in the food chain. Once the Pb enters the food chain, it causes biochemical, physiological, and behavioral abnormalities [15]. The Pb poisoning is both direct (through the intake of contaminated drinking water and food crops) and indirect (through the meat–milk route) when the animals are fed Pb-contaminated food or water and later consequently pass the residues of Pb to humans via meat and dairy products. As shown in Figure 2, Pb in the ecosystem may be transported to humans through the direct route (contaminated water and food) and the indirect route (meat–milk transfer from the contaminated animals).

Pb interrupts plant physiology by inducing oxidative stress, preventing chlorophyll synthesis, and inhibiting root elongation [16]. It also disrupts the microbial ecology by compromising soil fertility and nitrogen fixation [17]. Pb is a strong systemic toxin that is usually caused by ingesting contaminated water or food. However, in some cases, it is caused by accidental ingestion of contaminated dust or paint that was based on Pb, or even by the bloodstream [18]. It is a highly toxicant with no known safe level of exposure, especially for children (whether inhaled or swallowed) and for humans and animals. Pb creates neurotoxicity by interfering with neurotransmitter release, neuronal signaling, and synaptic plasticity. Long-term exposure to even low levels of Pb toxicity can lead to reduced IQ, learning disabilities, and behavioral problems in children [19]. In addition, long-term exposure to Pb may cause injury to the renal tubules and thereby impair kidney function, thus leading to gout [20]. Pb inhibits enzymes such as δ-aminolaevulinic acid synthase and ferrochelatase synthase, which are crucially involved in heme biosynthesis. Consequently, Pb toxicity causes anemia. It is also known to reduce the lifespan of red blood cells by increasing oxidative stress [18,21].

Pb damages the cardiovascular system, muscles, gastrointestinal, reproductive, immune, and nervous systems, and causes brain disorders both in children and adults [22,23]. Pb affects the strength of communication between cells and alters the circuitry of the neurons. In both soft tissues and bones, Pb causes blood disorders in mammals, affecting almost every organ and system in the body [24]. Long-term exposure to Pb or its salts can cause nephropathy, colic-like abdominal pains, miscarriage in pregnant women, reduced fertility in males, weakness in fingers, ankles, or wrists, severe damage to the brain and kidneys in adults or children, and ultimately death [25,26]. The sensitive population groups of Pb toxicity include pregnant women, fetuses, and children. Pb can cross the placental barrier to fetal exposure and influence the development of fetuses, resulting in low birth weight and neurodevelopmental delays [27].

### 1.2. Pb Removal Strategies

**Figure 3:**
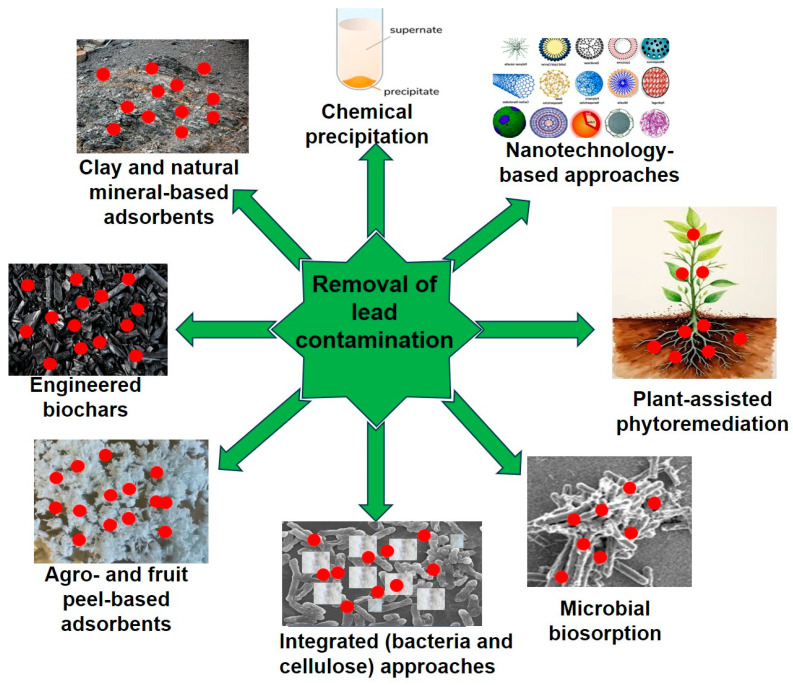
Pb removal strategies. A conceptualized summary of significant methods applied in the elimination of Pb contamination, which comprises chemical precipitation, microbial biosorption, nanotechnology-based treatments, plant-assisted phyto-remediation, integrated bacteria–cellulose systems, agro- and fruit peel-derived adsorbents, engineered biochar, and clay or natural mineral adsorbents. The red circles denote lead ions.

Several conventional methods for Pb removal from wastewater, including evaporation, solvent extraction, ion exchange, chemical precipitation, reverse osmosis, membrane filtration, electrochemical treatments, electrocoagulation, electrodialysis, and adsorption using synthetic materials, have been studied [28,29,30,31,32,33]. Under controlled laboratory conditions, these remediation methods can achieve comparatively high removal efficiencies. However, these methods are often associated with several limitations due to the generation of secondary waste pollutants in the form of sludge and the generation of toxic byproducts. The incomplete removal of trace-level heavy metals requires further treatment and proper disposal. In addition, the conventional treatments require the consumption of large amounts of chemical reagents and energy, skilled operation, sophisticated infrastructure, and high operational costs [2,34,35]. The membrane-based technologies are effective but tend to be quite expensive, prone to membrane fouling, and of high operating costs. Furthermore, these processes are not always suitable for treating wastewater containing low concentrations of Pb due to economic limitations and efficiency issues [36,37]. Thus, it is essential to successfully implement sustainable, low-cost, and eco-friendly techniques, particularly in rural and underserved regions where Pb pollution is prevalent. Figure 3 demonstrates several other crucial approaches, such as nanotechnology, phytoremediation, microbial remediation, agro-based adsorption, biochar, clay, and natural mineral-based adsorbents, which are widely used for the remediation of Pb from contaminated sources. These are eco-friendly strategies employed for the effective removal of Pb contamination from the environment.

Scientists all over the world have been progressively focusing on agriculture and biologically derived materials as promising substitutes for Pb remediation, such as microbial adsorption, phytoremediation, and bioaccumulation. Microbial biosorption takes advantage of the inherent potential of bacteria, fungi, and algae to adsorb and sequester heavy metals through cell wall functional groups, and it has shown high effectiveness in Pb^2+^ removal from aqueous solutions [38,39]. These microorganisms are highly specific with high flexibility to environmental factors and regenerative abilities, thus making them environmentally friendly for Pb clean-up procedures [40,41].

Similarly, phytoremediation approaches demonstrate the ability of certain macrophytes to stabilize, uptake, or volatilize heavy metals from contaminated water or soil via root systems, providing a low-cost alternative to physicochemical methods [42,43]. Cellulose-based adsorbents derived from various fruit peels or leaves constitute another potential category of biosorbents. There are many functional groups in fruit peel cellulose that possess a strong affinity to metal ions. At the same time, the development of nanotechnology has advanced the metal remediation industry by providing new materials with remarkable physicochemical characteristics like high surface area, distinct reactivity, and improved metal-binding capacity [44]. Nano-iron oxides, carbon nanotubes, metal–organic frameworks (MOFs), graphene derivatives, and nano-zeolites are characterized by excellent performance in the elimination of Pb in aqueous environments [38,45]. These materials interact with Pb^2+^ ions through an adsorption process, redox reaction, and surface complexation. In spite of regulatory obstacles, environmental persistence, and toxicity concerns, nanotechnology continues to be a frontier in the development of next-generation water purification systems [46].

### 1.3. Aim of the Study

Despite extensive research on Pb remediation using individual technologies, there is a lack of comprehensive reviews that integrate all significant green and advanced technologies. The present study aims to provide a complete and comparative analysis of the different methods employed for Pb remediation from wastewater, including microbial, agro-waste, plant-based, fruit peel, biochar, clays, and naturally occurring minerals, and nanotechnology-based methods. The performance measures, cost-effectiveness, removal mechanisms, benefits, limitations, scalability, sustainability, field applicability, and regeneration potential of each of the methods are given special consideration. The review introduces critical discourse of integrated and hybrid systems, their corresponding challenges, and research perspectives in the future.

The novelty of this review is that the innovations are integrated in the biological, agricultural, and nanotechnological fields to eliminate Pb in a single framework. Through assessment and comparison of a vast range of methods, the review aims to assist researchers, environmental engineers, and policy-formulating bodies in choosing and designing effective, sustainable, and context-appropriate remediation systems. This review is useful as an informative tool to environmental researchers, wastewater engineers, and policymakers in selecting effective and appropriate solutions for mitigating Pb contamination globally.

## 2. Review Methods

An extensive search of literature was conducted in the various electronic databases such as Web of Science, Science Direct, PubMed, PubMed Central, and Google Scholar of publications regarding Pb bioremediation between 2010 and 2025. A few studies published before 2010 were included when they were crucial to provide historical context or to substantiate important ideas. The literature search was conducted in September and October 2025. The search strategy used a combination of keywords that were relevant, i.e., lead remediation, microbial lead resistance, microbial methylation of lead, biochar, bio-based adsorbents, biological volatilization of lead, cost-effectiveness, fruit peels, microbial detoxification, nanotechnology, phytoremediation, hyperaccumulator plants, sustainable remediation, and wastewater treatment. All the obtained studies were initially screened by their title and abstract, and then the full text was evaluated in detail where necessary. Only peer-reviewed journal articles and book chapters published in English were considered qualified for addition. The exclusion criteria included the lack of full-text access, non-English, and non-peer-reviewed articles, such as letters to the editor and abstracts of conferences.

## 3. Microbial Approaches for Pb Remediation

Microorganisms develop complex mechanisms to survive in the adverse environment with metal ions when they are grown in the polluted environment, making them highly effective mediators for bioremediation of heavy metals, including Pb. Among the biological approaches for bioremediation, microbial bioaccumulation and biosorption have been developed as low-cost, sustainable, and environmentally friendly alternatives for Pb removal from wastewater [38,47]. This technique uses the intrinsic ability of microbial cells to bind, immobilize, or digest the inactive metal ions through active or passive processes [48]. Besides bioaccumulation and biosorption, other promising removal efficiencies and moderate or high adsorption capacities are shown by microbial-based technologies such as reduction, methylation, oxidation, and volatilization by bacteria, fungi, and algae [49,50,51]. Prithviraj et al. (2014) and Kondakindi et al. (2024) demonstrated that biosorption is a passive adsorption of metal ions onto the cell wall components, which are aided by functional groups (carboxyl, hydroxyl, phosphate, amine, and sulfhydryl) [5,52]. This process usually takes place in the living and dead biomass. Conversely, bioaccumulation is an active metabolism-driven process in which living cells take metal ions into their intracellular compartments. In most cases, they are held in vacuoles or bound to metallothionein and other intracellular ligands [53]. The effectiveness of microbial biosorption depends on many factors, such as the species of the microbes and the nature of the cell wall, as well as the physicochemical properties of an aqueous solution [38,48].

**Figure 4:**
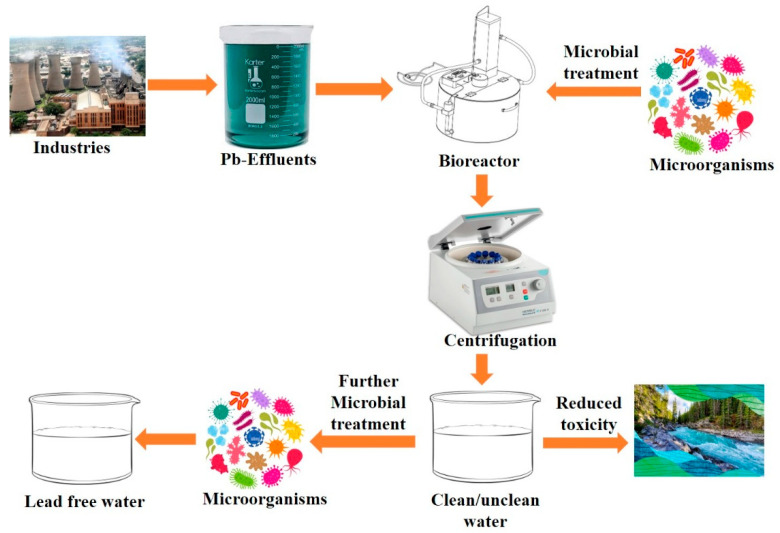
Microbial approaches to Pb remediation. The schematic diagram of the treatment of Pb-contaminated industrial effluent using the Pb-resistant microorganisms.

Different microbial communities have exhibited significant capability for Pb removal. Among bacteria, *Bacillus subtilis*, *Pseudomonas aeruginosa*, *Ralstonia metallidurans*, *Lysinibacillus*, *Streptomyces*, and *Escherichia coli* have been extensively researched [54,55,56,57]. These bacteria are highly metal-binding due to their cell walls made of peptidoglycans and the existence of extracellular polymeric substance (EPS), which enhances the adsorption of Pb. *Bacillus* species have been known to be resilient, easy to cultivate, and highly tolerant to heavy metals [56]. Figure 4 shows that effluents contaminated with Pb can be treated successfully in a bioreactor using Pb-resistant microorganisms to obtain cell-free water with greatly minimized Pb toxicity. Treated water is then assessed to have less Pb toxicity, and further processing of microbes can yield Pb-free water that can be safely discharged.

The filamentous types of fungi, particularly *Aspergillus niger*, *Penicillium chrysogenum*, and *Trichoderma harzianum*, among others, are known to have strong biosorption abilities [58]. They possess numerous metal binding sites, which are abundant in their cell walls containing a lot of chitin and glucan, and their hyphae structure provides them with a large surface area to interact with metal ions [59]. Additionally, these fungi can produce organic acids and chelators, which cause mobilization or immobilization of Pb, depending on environmental conditions [58]. Moreover, yeasts such as *Candida albicans* and *Saccharomyces cerevisiae* have been widely studied because of their ability to bioaccumulate metal ions [60].

Microalgae such as *Scenedesmus obliquus*, *Chlorella vulgaris*, and *Spirulina platensis* have been widely researched concerning their ability to sequester Pb in the aqueous media [61,62]. Their cell walls are made up of polysaccharides and proteins, and have negatively charged functional groups that pull in positively charged Pb^2+^ ions [59,63]. Further, the ability to grow in nutrient-rich wastewater makes them suitable for combined wastewater treatment systems [64]. Algae can be easily and efficiently harvested and used for biomass production after metal extraction, thereby aiding in sustainable waste management. Thus, it can serve as an important feedstock in biofuel production and fertilizer manufacture, therefore supporting circular economy strategies [65,66].

Also, the efficiency of microbial Pb removal is strongly influenced by many environmental and operational parameters, such as pH, temperature, initial metal concentration, contact time, nutrient availability, and the physiological condition of the microorganisms. These are among the most crucial parameters, since they influence both the ionization state of functional groups on the cell surface and the solubility of Pb^2+^ ions [67,68]. The optimal pH for biosorption of Pb often falls between 4.0 and 7.0. Among the metals tested, Pb was found to have the greatest biosorption efficiency (over 95 percent at pH 7) because of its high affinity with other functional groups on the surface biomass, such as the carboxyl and hydroxyl groups [67]. When the pH is low, the high concentration of H^+^ and H_3_O^+^ protonates the hydroxyl and carbonyl groups, which bind to the sites with aqueous heavy metal ions in biosorbents, resulting in the least or no metal adsorption. On the other hand, at higher pH, metal hydroxides may precipitate, limiting the biosorption [2,69].

Biosorption greatly depends on temperature. Even small increases tend to optimize it by increasing the diffusion rates and kinetic energy, although the cell wall structures become denatured at too high temperatures [70]. Higher initial biomass doses tend to give a greater number of binding sites, but can also be prone to aggregation and thus reducing the surface area [35]. Similarly, contact times of 30–120 min are usually appropriate to reach equilibrium, although the time is not constant depending on bacterial species and state of biomass (live or dead) [64].

Despite having many advantages, microbial bioremediation methods are not without limitations. Living biomass is susceptible to metal toxicities and environmental stress, potentially unfavorable for growth and survival in very polluted waters [71]. On the other hand, biosorption on dead biomass alleviates the toxicity issues but does not allow regeneration or adaptation to changing environmental conditions. In addition, isolation and recycling of microbial biomass may be a challenge, particularly in continuous-flow treatment systems [71]. The possible discharge of microbial metabolites and organic substances into the treated water requires careful attention, particularly in large-scale applications. However, these limitations can be mitigated using immobilization techniques, genetic modification, and process optimization [72].

Recent case studies have proved that microbial Pb remediation is practically useful. A study by Pal et al. (2025) used *Enterobacter chuandaensis* live biomass of DGI-2 to eliminate Pb^2+^ from wastewater with a removal percentage of 94.73 under optimized conditions [73]. In a different work, a maximum Pb adsorption concentration of 10,000 ppm was demonstrated by *Bacillus amyloliquefaciens*, which was extracted from industrial effluents [74]. In the same manner, *Bacillus cereus* SEM-15 demonstrated a high adsorption capability of 150 mg/L, achieving over 93% elimination efficacy [75]. Cyanobacteria that fix nitrogen, such as *Anabaena* sp. and *Nostoc muscorum*, possess a high degree of efficiency in the remediation of Pb-contaminated water. *Anabaena* sp. and *N. muscorum* were recorded to have efficiencies of 98.90 and 88.00 towards Pb, respectively [50].

The genetically modified bacterial strains, including *E. coli*, which encode metal-binding proteins, have the potential to significantly increase the Pb biosorption efficacy [76]. The high-affinity metal-binding peptides of engineered microorganisms, CRISPR-based strain enhancement, and co-culturing of synergistic microbial consortia are under research to enhance Pb uptake, tolerance, and system resilience [77]. Hence, microbial remediation approaches offer an effective and environmentally friendly alternative solution for Pb remediation in wastewater, particularly when improved and integrated with other technologies. Their elevated surface reactivity, adaptability, and cost-effectiveness make them suitable for centralized and decentralized water treatment systems. The challenges of microbial physiology, genetic modification, and process engineering should be investigated further to address current challenges. Table 1 highlights the general description of the prominent microorganisms used in the treatment of Pb-contaminated wastewater, highlighting their individual removal modes reported in earlier studies.

## 4. Agro-Waste- and Fruit Peel-Based Adsorbents for Pb Remediation

The rising environmental effects of Pb pollution in water have caused significant research on low-cost sustainable adsorbents, mainly agro-waste and fruit peels, which have gained considerable attention. These biomass materials are easy to obtain, renewable, and exhibit significant adsorption capacity due to their large number of functional groups, e.g., hydroxyl groups, carboxyl groups, and phenolic groups [92,93]. These functional groups are capable of reacting with Pb^2+^ ions through complexation, ion exchange, and surface adsorption, and thus, agro-waste-based adsorbents can be used in the removal of Pb in contaminated water [94].

**Figure 5:**
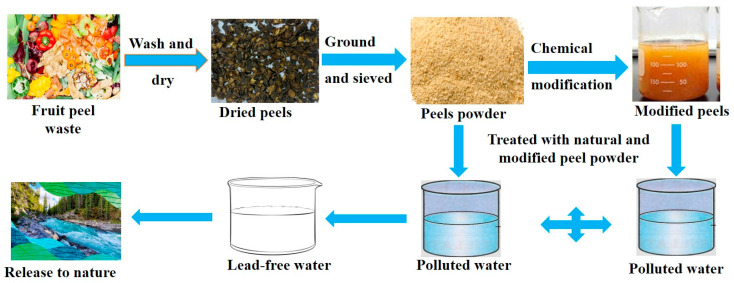
Schematic illustration of the process of Pb remediation with agro-waste and fruit peel-based adsorbents. The peels were dried, ground, and chemically modified to increase adsorption competence. The chemically modified peel powder is then used on Pb-contaminated effluent, and the treated cell-free water is evaluated to determine how much toxicity of Pb is removed.

Various agro-waste materials, among the most studied cellulose-based adsorbents derived from agricultural by-products that have been used for treating industrial effluents are apple peel [95], banana peel [96], corncob [97], coconut shell [98], lemon peel [99], orange peel [100], watermelon rind [101], rice husk [97,102], moringa seeds [103], wheat husk [104], sawdust [105], peanut husk [106], potato peel [96], sunflower biomass [107], sugarcane bagasse [108], waste tea leaves [109], marine algal biomass [110], shrimp shells [2,35], water hyacinth [111], neem leaf [112], etc. A schematic diagram demonstrates the preparation and use of agro-waste and/or fruit peel cellulose to remove Pb from polluted water (Figure 5). These agro-materials are used in either natural forms or chemically modified ones in order to increase their adsorption properties. Lignocellulosic materials are rich with cellulose, hemicellulose, and lignin with functional groups having a capacity to bind Pb^2+^ ions through ion exchange, chelation, and electrostatic interactions. Their uses reduce the waste management cost and raise the value of agricultural residues, as they can be used as useful adsorbents [113]. Fruit peel cellulose that is chemically modified enhances selectivity and adsorption potential, and its use is aligned with the approach for the circular economy that combines the use of waste reuse and reductions in pollution [114]. The utilization of such waste products offers an economic solution to the cleanup of Pb and, at the same time, addresses the challenge of solid waste management by means of sustainable utilization of their agricultural by-products. Physical, chemical, or biological modifications can be successful in enhancing the adsorptive property of untreated agro-waste and peels of fruits. Surface area and active sites are enhanced with the help of chemical activation, which can be carried out with the help of acids, bases, or metal salts [115]. Like microbial remediation, the effectiveness of adsorption using agro-waste depends on several factors such as pH, contact time, initial Pb concentration, temperature, and type of adsorbent [116]. Pb absorption is more efficient at neutral to acidic pH because of the characteristics of the adsorbent surface charges. Several studies have shown promising findings, such as orange peel cellulose that has exhibited Pb removal efficiencies of 98.33% under ideal conditions [100]. Apple peel beads exhibited elevated adsorption capacities of 73% [95]. Powdered and beaded lemon peel adsorbent materials had high Pb removal efficiencies over 86% [99]. The kinetics of Pb adsorption on these substrates typically follow a pseudo-second-order model, indicating that chemisorption is the predominant mechanism, while isotherm investigations are often characterized by Langmuir models, suggesting monolayer adsorption. Generally, fruit peel- and agro-waste-based adsorbents are prospective, sustainable materials for Pb removal in water systems [117]. Their low cost, eco-friendliness, and the ability to bind Pb effectively makes them an attractive alternative to the conventional adsorbents.

Although the use of agro-based cellulose in the removal of Pb bioremediation has demonstrated benefits, there are several limitations associated with the use of this technology. Challenges exist in the regeneration and reuse of agro-waste adsorbents, due to the risk of structural degradation or the inability to desorb the Pb ions. Additionally, adsorption consistency may be influenced by variations in composition due to geographic origin, season, and processing technique. However, continuous studies on composite materials combining agro-waste and nanomaterials or polymers to enhance stability, adsorption ability, and selectivity [118]. Municipal systems have reported removal efficiencies that are usually based on the concentration of Pb in influent, pH control, as well as the treatment technology used. The combination of laboratory and municipal data would provide a more comprehensive understanding of the effectiveness of Pb^2+^ remediation at large scales [119]. The pilot-scale and optimization studies are necessary to establish uniform preparation procedures of those systems, enabling real-life applications of these technologies in wastewater treatment and environmental remediation [120]. Table 2 shows selected agro-waste and fruit peel-derived adsorbents prepared for the remediation of Pb, their modification strategies, adsorption, and optimal conditions documented in the literature with their references.

## 5. Phytoremediation-Based Strategies

Phytoremediation is an eco-friendly and sustainable method for mitigating Pb pollution using plants. It is one of the most used methods, particularly in the case of massive land cleanup. The principle of this strategy involves taking advantage of the natural capacity of some plants to absorb, accumulate, convert, or stabilize heavy metals, such as Pb, in soils or water [131]. Due to its low cost and lesser environmental impact, phytoremediation appears to have very high potential in mass and long-term Pb cleanup of both urban and rural environments [132].

**Figure 6:**
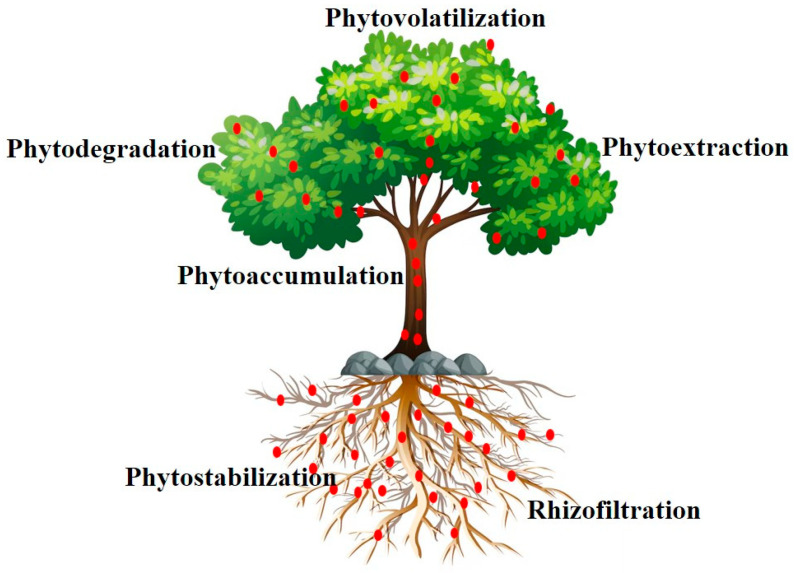
Schematic representation of Pb-accumulating plants used for the remediation of soil and water contamination. Plants uptake heavy metals via their roots and translocate them to the different parts, where the metals can be extracted, degraded, or volatilized. Red spots indicate Pb^2+^ ions.

There are several mechanisms by which phytoremediation can remove or neutralize Pb contamination (Figure 6). In phytoextraction, the Pb contamination in soils is absorbed by plants and transported to the plant shoots, which are then removed through harvesting [133]. On the other hand, phytostabilization limits the mobility of Pb in the soil by trapping it in the rhizosphere or attaching it to plant roots [134]. Rhizofiltration uses roots to absorb Pb in aqueous solutions [135]. Phytovolatilization is less common with Pb and incorporates transformation to volatile compounds, and is usually used on other metals. Phytodegradation is the process by which plant enzymes break down organic contaminants [136]. However, it plays a limited role in Pb detoxification.

Some hyperaccumulator plant species, such as *Brassica juncea* (Indian mustard) [137], *Cannabis sativa* (hemp) [138], *Helianthus annuus* (sunflower) [139,140,141], and *Vetiveria zizanioides* (vetiver grass) [142], are the most well-known for effective Pb removal. These species have a high biomass and can tolerate high levels of Pb. Under optimal conditions, Indian mustard can store more than 1000 mg Pb/kg dry weight. Sunflower plants and vetiver can be effective in extracting large amounts of Pb in soil and water because of their deep root system and adaptation to metal stress. Table 3 provides a comparative analysis of different phytoremediation strategies employed in Pb-contaminated environments, including the plant species, mechanism, efficiency, and references.

The effectiveness of Pb removal by plant species is highly dependent on the environmental parameters, such as organic matter content, soil pH, Pb speciation, and the presence of chelating agents such as EDTA and citric acid [143]. It has been suggested that phytoextraction with the help of chelators can enhance the uptake and bioavailability of Pb, though it raises environmental concerns because of the potential leaching of soluble Pb complexes.

Despite its promise, phytoremediation has some limitations, such as the process being slow and seasonal, often requiring several years for effective remediation [43]. Additionally, proper disposal and careful handling of contaminated biomass are required to prevent secondary pollution [144]. Nevertheless, phytoremediation can be incorporated with other techniques, like soil amendments and microbial consortia, which can improve the performance and broaden applicability. Genetic engineering and plant-microbe interactions, such as plant growth-promoting rhizobacteria, are being actively studied to improve Pb uptake and removal from contaminated sources.

**Table 3:** Comparative overview of strategies of phytoremediation-based Pb removal, including the names of plant species, plant type, strategy, mechanisms, efficiencies of Pb removal, and references.

Plant Species	Type	Strategy Type	Mechanism	Pb Removal Efficiency	Reference
*Eleusine indica*	Herbaceous annual grass	Phytostabilization	Uptake into shoots	7474 mg kg^−1^	[145]
*Lactuca sativa*	Annual herb (Lettuce leaves)	Biosorption	Leaves	71.22 mg/g (89.02%)	[146]
*Eichornia crassipes*	Water hyacinth	Rhizofiltration	Uptake into roots	92.4%	[111]
*Cannabis sativa* L.	Industrial hemp	Phytoextraction	Uptake into roots	>100 µg/g	[138]
*Helianthus annuus*	Sunflower	Phytoextraction	Uptake into roots and shoots	410 mg/kg (roots), 180 (shoots)	[141]
*Vigna unguiculata*	Cowpea	Phytoextraction	Uptake into roots	58.1 mg/kg DW	[139]
*Brassica pekinensis*	Chinese cabbage	Phytoextraction	Uptake into roots	50.0 mg/kg DW	[139]
*Gomphrena globose*	Globe Amaranth	Phytoextraction	Uptake into roots	25.7 mg/kg DW	[139]
*Helianthus annuus*	Sunflower	Phytoextraction	Uptake into roots	23.5 mg/kg DW	[139]
*Limbarda crithmoides*	Sunflower	Bioaccumulation	Uptake into roots and shoots	906.2 mg/kg (roots), 474.2 (shoots)	[140]
*Helianthus annuus*	Sunflower	Bioaccumulation	Uptake into roots and shoots	887.9 mg/kg (roots), 256.2 (shoots)	[140]
Hydrangea	Endless summer	Phytoextraction	Uptake into roots and shoots	823.39 ± 163 mg/kg (roots), 81.11 ± 7.16 (shoots)	[147]
Hydrangea	Flowering plants	Phytoextraction	Uptake into roots and shoots	408.13 ± 123.79 mg/kg (roots), 69.53 ± 7.18 mg/kg (shoots)	[147]
Hydrangea	Ayesha	Phytoextraction	Uptake into roots and shoots	700.89 ± 44.59 mg/kg (roots), 93.86 ± 11.94 mg/kg (shoots)	[147]
*Cyamopsis tetragonoloba*	Cluster bean	Bioaccumulation	Uptake into roots, stems, and leaves	336.92 mg/kg (roots), 124.19 mg/kg, (stems), 47.45 mg/kg (leaves)	[148]
*Hedera colchica*	Evergreen climbing plant	Bioaccumulation	Uptake into roots and shoots	252 mg/kg (roots), 92.2 mg/kg (shoots)	[149]
*Phyllostachys* *pubescens*	Moso bamboo	Bioaccumulation	Uptake into roots, stems, and leaves	4282.8 mg/kg (roots), 482.2 mg/kg, (stems), 148.8 mg/kg (leaves)	[150]
*Plantago major* L.	Perennial non-woody herb	Bioaccumulation	Uptake into roots	9284.66 mg/kg	[151]
*Miscanthus floridulus*	Rhizomatous grass (herbaceous plant)	Bioaccumulation	Uptake into roots and shoots	214.8 mg/kg (roots), 109.2 mg/kg (shoots)	[152]
*Saccharum officinarum* L.	Sugarcane	Phytoextraction	Uptake into roots and shoots	1750 mg/kg (roots), 1250 mg/kg (shoots)	[153]
*Brassica juncea* L.	Indian Mustard	Bioaccumulation	Uptake into roots	79.2 mg/kg	[137]
*Koelreuteria paniculata*	Deciduous ornamental tree	Bioaccumulation	Uptake into roots, stems, and leaves	3187.87 ± 251.77 mg/kg (roots), 389.46 ± 21.7 mg/kg (stems), 253.11 ± 7.81 mg/kg (leaves)	[154]
*Zea mays*	Maize	Phytoattenuation	Uptake into roots and shoots	182.3 ± 9.9 mg/kg (roots), 25.8 ± 4.4 mg/kg (shoots)	[155]

## 6. Biochar and Activated Carbon-Based Technologies

Biochar and activated carbon are useful adsorbents in removing Pb particles from polluted water due to their high surface area, porous structure, and abundance of functional groups [156]. Both of these materials are carbonaceous and often derived from biological biomass. They differ in their production and physicochemical properties. They are usually prepared by pyrolysis from agricultural or forestry residues in a limited oxygen supply. Biochar is attractive due to its sustainability and cost-efficiency, especially when produced from agricultural waste materials like rice husk, coconut shell, peanut shell, pinewood, tea waste, or maize stover [157]. Oxygen-containing functional groups (-OH, -COOH) contribute to better Pb binding by surface complexation and ion exchange [158]. The methods of modification, including impregnation with metal oxides, such as Fe, Mn, and Zn, or chemical activation using KOH and H_3_PO_4_, have a significant effect on the sorption performance of biochar [159]. The use of iron-impregnated biochar can also enhance the Pb removal because of the development of inner-sphere interactions between Pb^2+^ and the Fe-O functional groups [160]. Surface modification facilitated the ion exchange process, increasing the content of Mg^2+^, thus providing Pb^2+^ with an increasing number of exchange sites. This augmented surface Mg^2+^ has been a decisive factor in the overall adsorption process since it enhanced the exchange of ions with Pb^2+^. Besides ion exchange, surface complexation of Pb^2+^ ions with the oxygen-containing functional groups (i.e., -COOH, -OH) on the HCC-Mg surface also plays a role in the removal of Pb [161].

Activated carbon is produced by means of physical or chemical activation, particularly by the granular or powdered type, which has a greater surface area and better pore structure, leading to a greater adsorption capacity [162]. Activated carbon is commonly processed from coconut shells, wood, and coal, and it is used in commercial water treatment systems [163]. Activated carbon has high microporosity, which promotes physical adsorption of Pb ions, whereas the surface chemistry, especially after chemical treatment, enables chemisorption through electrostatic forces of attraction, precipitation, and complexation [164]. Modified activated carbon materials, such as aminated or magnetic variants, are more selective and can be recovered after use [165].

Previous studies demonstrated that the activated carbon and biochar showed Pb adsorption capacity over 700 mg/g at the optimal conditions [166,167,168]. The adsorption kinetics typically follow pseudo-second-order models. Nevertheless, isotherm analysis is usually consistent with Langmuir/Freundlich models, which reflect monolayer or heterogeneous adsorption on the surfaces. Both materials show high regeneration potential through acid or alkaline desorption, facilitating numerous reuse cycles without loss of efficiency. Hence, biochar and activated carbon technologies deliver an efficient, scalable, and adaptable solution to Pb cleanup. Though activated carbon offers high performance, biochar is a more sustainable and economically viable substitute, particularly in rural and resource-limited contexts [164]. Future research should focus on optimizing production, developing hybrid materials, and assessing the long-term field effectiveness in the removal of Pb safely and efficiently. Several studies show that lignocellulosic feedstocks such as watermelon rind, pinewood, rice straw, and corncob residues consistently exhibit higher Pb adsorption capacities (often >500 mg/g), especially when combined with suitable chemical modifications. Table 4 summarizes the performance of the different biochar and activated carbon adsorbents in the Pb remediation with a focus on their production methods, modification techniques, and the reported adsorption capacity. From Table 4, it is observed that Fe impregnation, MnOx loading, MgO addition, and KOH activation significantly enhance adsorption capacity by introducing active sites and improving surface chemistry. It is also observed that higher pyrolysis temperatures (e.g., 700–900 °C) increase surface area and aromaticity, improving Pb sorption (e.g., 700 °C watermelon rind biochar: 742 mg/g). On the other hand, lower temperatures tend to preserve more oxygen-functional groups, promoting ion exchange and surface complexation.

However, using biochar and activated carbon for Pb removal still has some limitations. The preparation of activated carbon is energy-demanding, and the cost might be high in resource-limited environments [166]. On the other hand, raw biochar may exhibit reduced adsorption capacity and inconsistent performance due to feedstock variability [167]. Environmental concerns regarding the possibility of leached residual pollutants of the biochar need to be overcome by post-treatment and quality measures.

**Table 4:** Comparison and summary of biochar- and activated carbon-based adsorbents in the removal of Pb, with respect to the source, pyrolysis temperature and duration, modification/treatment, maximum Pb adsorption/removal capacity, highlights, and references.

Feedstock/Source	Pyrolysis Temp; Time	pH; Initial Pb Conc.	Modification/Treatment	Maximum Pb Adsorption/Removal Capacity (mg/g)	Key Highlights	Reference
Rice straw	420 °C; 4 h	5.0; 0.5 mmol	KMnO_4_	305.25 mg/g, (90%)	MnOx showed high sorption capacity to Pb(II)	[169]
Green waste (GWB)	650 °C	9.3–10.6; 1.6–7.0 mg/kg	GWB (pH = 9.3) with Fe caused a decrease in their pH to 3.4	736 mg/g (92.9%)	Precipitation, surface complexation	[167]
Shell	200 °C; 8 h	6.0; 50 mg/L	FeCl_3_·6H_2_O, EDTA	129.31 mg/g	Synthetic biochar (BC), magnetic biochar (M-BC), and EDTA functionalized magnetic biochar	[170]
Pinewood sawdust	350 °C; 1 h	7.0; 100 mg/L	Al(NO_3_)_3_·9H_2_O, MgSO_4_·7H_2_O	591.20 mg/g	Complexations and electrostatic attraction	[171]
Eucalypts leaf	700 °C; 2 h	7.0 ± 0.05; 100 mg/L	Modified using ZnCl_2_, FeCl_3_ and FeSO_4_	52.40 mg/g, 84.1%	EDTA-2Na was effective in desorbing Pb(II) and regenerating the adsorbent.	[172]
Watermelon rind	700 °C; 1 h	10.49–10.72; 50 mM	MgO	742 mg/g	Strong potential for environmental remediation	[168]
Douglas fir	900–1000 °C; 10 s	5.0; 100 mg/L	Modified using KOH	140 mg/g	KOH activation remarkably increased the surface area from 535 to 1050 m^2^/g	[173]
Palm fiber	400 °C; 2 h	6.5; 100 mg/L	FeSO_4_·7H_2_O and FeCl_3_·6H_2_O	188.18 mg/g (>97.9%)	Biochar showed a high removal rate, selectivity, separation, and reusability for Pb (II)	[174]
Pine wood	600 °C; 1 h	5.5; 50 mg/L	Modified using MnCl_2_·4H_2_O	47.05 mg/g	Modifications were used to improve sorption ability	[175]
Hickory wood	600 °C; 1 h	6.0–7.0; 100 mg/L	KMnO_4_	153.10 mg/g	Dosage, initial solution pH, and affected heavy metal removal	[176]
Hickory wood	600 °C; 2 h	5.0; 100 mg/L	NaOH	53.60 mg/g	Modification enhanced surface area, cation-exchange capacity, and thermal stability	[177]
Swine manure	450 °C	5.85; 228 mg/kg	The fresh swine manure was dried at 105 °C for 24 h before pyrolysis	228 mg/g (92%)	Precipitation, ion exchange, π bond action	[178]
Crofton weed		5.0–6.0; 200 mg/L	Modified using MgO	384.08 mg/g	An efficient and low-cost MgO-biochar for Pb^2+^/Cd^2+^ removal	[179]
Rice straw	550 °C; 2 h	5.0; 1 mmol/L	Not further modified	176.12 mg/g	Higher pyrolysis temperature had higher affinities due to enhanced surface area	[180]
Corncob-to-xylose residue	400 °C; 2 h	5.00 ± 0.05; 100 to 500 mg/mL	Nitrogen doped magnesium oxide	1429 mg/g	Ion exchange, precipitation, and complexation	[166]
Rice husk, wheat straw, and corncob	550 °C	5.5 ± 0.5; 1.95 mg/mL	Not further modified	96.41%, 95.38%, and 96.92%	Environmentally friendly adsorbent materials for energy-efficient, cost-effective, and cleaner water production	[97]
Corn stalks	800 °C; 2 h	6.0; 200 mg/L	Nanoscale zero-valent iron, KOH	480.9 mg/g	nZVI-HPB nano-composites present superior performance for Pb^2+^ removal	[181]
Pomelo peel	250 °C; 2 h	≈6.0; 50 mg/L	H_3_PO_4_	88.70 mg/g	Adsorption via chemical reduction and precipitation	[182]
*Quercus robur*	250 °C; 4 h	6.8 ± 0.5; 100 mg/L	Modified with FeCl_3_ and FeCl_2_	63.60 mg/g	Pb is removed by electrostatic interactions	[183]
Rice husk	800 °C; 3 h	5.0 ± 0.1; 250 mg/L	Manganese oxide	86.50 mg/g	Adsorption was due to the existence of π-electrons and surface OFGs	[184]
Rice husk	300 °C; 2 h	5.0–6.0; 100 mg/L	β-cyclodextrin	240.13 mg/g	Electrostatic attraction and complexation	[185]
Biogas residue	700 °C; 2 h	5.0; 50 mg/L	FeCl_3_, FeSO_4_·7H_2_O	131.24 mg/g	Complexation and precipitation on the surface, with electrostatic attraction	[186]
Water hyacinth	433 °C; 160 min	5.42 ± 0.03; 50 mg/L	Modified through optimized pyrolysis conditions	251.39 mg/g	Precipitation, surface adsorption, and functional group complexation	[187]
Date seed	550 °C; 3 h	6.0 ± 0.1; 4.0 mM	HCl	188.55 mg/g	Surface-modified biochar improved Pb, copper, and nickel removal	[188]
Peanut shell	400 °C; 1 h	6.5; 20 mg/L	Hydrated manganese oxide	330 mg/g	The adsorbent was fully regenerable without capacity loss	[189]
Sludge	600 °C; 90 min	6.0; 100 mg/L	Persulfate-zvi	180 mg/g	Ion exchange, reduction, and electrostatic attraction	[190]
Swine sludge	300 °C; 30 min	5 ± 0.3; 100 mg/L	Thiourea	145 mg/g (32%)	Adsorption rate ~5–8 times higher than unmodified biochar	[191]

Notes: Adsorption capacities depend on pH (typically ~5–6), initial Pb concentration, contact time, and surface chemistry. Chemical modifications like Fe impregnation or acid/alkali activation enhance adsorption by introducing active sites.

## 7. Nanotechnology-Based Approaches

Nanotechnology provides superior and effective solutions to Pb^2+^ remediation because of the superior surface area, reactivity, and tunable surface chemistry of nanomaterials [38]. These materials have improved adsorption rates and selectivity to Pb^2+^ ions, and frequently, they have higher speed and efficiency than conventional adsorbents [44]. Surface complexation of nanomaterials with Pb^2+^, ion exchange, electrostatic attraction, and redox reaction are possible depending on their composition and surface modifications [192].

Magnetite (Fe_3_O_4_) is one of the most popular nanomaterials that have been studied to date for the removal of Pb and is commonly applied in the form of magnetic nanoparticles (MNPs). Fe_3_O_4_ has excellent magnetic characteristics that enable simple recovery by magnetic separation, whereas its surface functional groups have the potential to bind Pb [192]. It is further functionalized with materials such as polyethylene glycol (PEG), humic acid, or chitosan, which enhance its adsorption capacity and stability in dispersion [193].

Another common nanomaterial that has been used is titanium dioxide (TiO_2_) nanoparticles. TiO_2_ nanoparticles are known to have photocatalytic properties, which allow the reduction and immobilization of Pb^2+^ under UV light [194]. Nonetheless, surface functionalization or integration into nanocomposites can also be used to increase their adsorption ability. Carbon-based nanomaterials, such as carbon nanotubes (CNTs), carbon graphene oxide (GO), and carbon quantum dots (CQDs), have shown excellent potential for the adsorption of Pb^2+^ ions [195]. They interact with Pb^2+^ ions strongly due to their high oxygen-containing functional groups and their great surface area. The selectivity and binding affinity can be further optimized by the modification of CNTs or GO with amino, thiol, or carboxyl groups [196].

Nano-zeolites, with their homogenous pore framework and good capacity to perform cation exchange, have also been applied in the efficient removal of Pb^2+^ in aqueous media. They are highly thermally stable and ion-exchange active and are therefore attractive, especially in composite forms or hybrid materials [197]. Hybrid nanocomposite metabolites that integrate metal oxides, carbon nanostructures, and biopolymers (e.g., chitosan, alginate) are used to enhance the properties of their constituents to improve the kinetics of sorption, mechanical stability, and regeneration. For example, the Fe_3_O_4_@GO or TiO_2_@chitosan nanocomposites possess synergistic properties in adsorption and reusability [198]. Table 5 delivers various nanomaterial-based methods of Pb^2+^ removal and emphasizes their working methods, adsorption characteristics, and significant benefits mentioned in the literature.

Although the nanotechnology-based approaches have better removal performance, they still have many challenges [199]. Toxicity and environmental risks of nanomaterials, particularly when being used in open systems, raise concerns about the long-term fate of these materials, bioaccumulation, and effects on aquatic systems. Also, in most areas, there are still regulatory frameworks that regulate the environmental use of nanomaterials [199]. The cost of preparation, environmental stability, and scalability are important considerations for practical applications. Thus, the nanotechnology methods may be considered as a front line of the Pb remediation, providing unparalleled effectiveness and flexibility. Nevertheless, safe design, sustainable synthesis, and responsible deployments play an important role in utilizing their full potential without unintended effects on the ecology.

**Table 5:** Comparison of nanotechnology-based strategies of Pb^2+^ removal, such as nanomaterials or nanocomposites, composition/type, modes of modification, and adsorption capacity or percentage removal, major strengths, and their reference list.

Nanomaterial/Composite	Composition/Type	Modification/Functionalization	Pb^2+^ Adsorption/Removal (%)	Key Advantages	Reference
Functionalized GOCA beads	Graphene oxide	Polyethylenimine modified graphene oxide	602 mg/g	Enhanced adsorption capacity, high efficiency and selectivity, good reusability	[200]
MgO nanoparticles	Metal oxide nanoparticles	No surface modification	148.6 mg/g	Adsorption + precipitation (MgO)	[201]
GO/PAMAMs composite	Graphene oxide/polyamidoamine dendrimers	Grafting to the GO/PAMAMs composite	568.18 mg/g	High Pb^2+^ adsorption capacity, fast equilibrium (within 60 min)	[202]
MnO_2_@Fe_3_O_4_/PmPD core	Magnetic Fe_3_O_4_ nanoparticles coated with poly(m-phenylenediamine) and MnO_2_ shell	MnO_2_ formed via redox reaction between KMnO_4_ and PmPD	438.6 mg/g	Electrostatic attraction, ion exchange, magnetically separable, and regenerable	[203]
CS/GO-SH composite	Chitosan/Sulfydryl-functionalized graphene oxide	Covalent modification (diazonium process) and electrostatic self-assembly with chitosan	447 mg/g	Improved structural properties, enhanced surface area	[204]
Polypyrrole-polyaniline/Fe_3_O_4_	Magnetic Fe_3_O_4_ nanoparticles	Surface coating with conducting polymer nanocomposite (PPy–PAn)	243.9 mg/g (up to 100%)	High Pb^2+^ removal efficiency, magnetically separable, regenerable with HCl/HNO_3_	[205]
MMSP-GO composite	Polyethylenimine-modified magnetic mesoporous silica with graphene oxide	Amine groups conjugated with GO carboxyl groups	333 mg/g	High Pb^2+^ adsorption, strong affinity due to amine-carboxyl interactions	[206]
CNC-g-BA	Cellulose nanocrystals from banana fiber	Grafting with butyl acrylate monomer	140.95 mg/g	Eco-friendly bio-based adsorbent	[207]
GO/MnFe_2_O_4_ nanohybrid	Graphene oxide with manganese ferrite (MnFe_2_O_4_) magnetic nanoparticles	Hybridization of GO with MnFe_2_O_4_ NPs	673 mg/g	Reusability, fast kinetics, large surface area, low-cost	[208]
EDTA-mGO composite	EDTA functionalized magnetic graphene oxide	Metal chelation + magnetic Fe_3_O_4_ incorporation	508.4 mg/g	Rapid magnetic separation (25 s), good reusability, spontaneous and endothermic adsorption process	[209]
GO/L-Trp composite	L-Tryptophan functionalized graphene oxide	Nucleophilic substitution reaction (GO functionalized with L-tryptophan)	222 mg/g	Fast sorption, exothermic, and spontaneous process, reusable for multiple cycles	[210]
Ze-nWTR	Zeolite + nano-drinking water treatment residuals (nWTR)	Composite formation of zeolite with nWTR	198.7 mg/g	High affinity for Pb^2+^, reusable, cost-effective, and sustainable	[211]
NH_2_–SG and NH_2_–SNHS	Amino-functionalized silica gel and silica nano hollow spheres	NH_2_ modification of SG and SNHS	96.79 mg/g	High affinity for heavy metals (Cd^2+^, Ni^2+^, Pb^2+^), monodisperse shape and size	[212]
Sil-Phy-NPANI	Nanosilica functionalized with nanopolyaniline	Green functionalization of nanosilica with PANI	186 mg/g	Efficient complexation/ion exchange via surface –NH_2_ and –OH groups	[213]
Sil-Phy-CrossNPANI	Nanosilica crosslinked nanopolyaniline	Immobilization via amine/hydroxyl groups	300 mg/g	Very high Pb^2+^ adsorption capacity; fast equilibrium (15–20 min)	[213]
1,4-phenylne diisocyanate (LPDIC)	Polymers synthesized from olive industry liquid waste (OILW)	Formation of urethane-linked polymeric foams from OILW organic components	20.86 mg/g	Biobased, cost-effective, sustainable, and with multiple binding sites	[214]
Maghemite nanoparticles (c-Fe_2_O_3_)	Iron oxide nanoparticles	Single-step synthesis	68.9 mg/g	High selectivity for multiple metals	[215]
MAMNPs	Maghemite (γ-Fe_2_O_3_) nanoparticles	Modification with homopolymers of mercaptoethylamino monomer	118.51 mg/g	Strong affinity for multiple heavy metals	[216]
IIP-MMT	Montmorillonite substrate with polymeric imprint. Surface ion imprinting via AGET-ATRP	Incorporation of PHEMA brushes and SHA chelating ligand	158.68 mg/g	Fast adsorption, strong stability, and reusability	[217]
Fe_3_O_4_/C	Magnetite (Fe_3_O_4_) nanoparticles	Integration of Fe_3_O_4_ with carbon	123.5 mg/g (99.83%)	Fast kinetics (30 min), spontaneous adsorption, reusable with high adsorption in multiple cycles	[218]
MnO_2_/gelatin composite	Dumbbell-shaped MnO_2_ nanoparticles with gelatin matrix	immobilization on an amino-modified PMMA plate	318.7 mg/g (83–100%)	Excellent stability and reusability; easy operation and practical application	[219]
Fe_3_O_4_@PTMT	Magnetic nanoparticles (MNPs)	Surface modification with organodisulfide polymer (PTMT)	533.13 mg/g	Rapid magnetic separation (20 s), recyclable up to 5 cycles	[220]
ZnONPCS	ZnO nanoparticles (~10 nm)	Biogenic synthesis using casein as a reducing and capping agent	194.93 mg/g (90%)	good regeneration and reusability, photocatalytic degradation of dyes	[221]
HFO-P(TAA/HEA) hybrid adsorbent	Hydrous ferric oxide (HFO) nanoparticles supported on porous polyhydrogel	In situ precipitation of HFO onto hydrogel matrix	303.8 mg/g	High selectivity for Pb^2+^ over competing ions	[222]

## 8. Clay and Natural Mineral-Based Adsorbents

Naturally occurring minerals and clays have been used as inexpensive, readily available, and less harmful substances for the adsorption of heavy metals, such as Pb^2+^, present in polluted water and soil. Their large mass availability and cation exchange capacities render them useful in large-scale uses, especially in developing countries [223]. The characteristic properties of the clay minerals include the high specific surface area and diverse structures, which is a reason to consider them as the most suitable in the manufacture of adsorbents [224].

The most investigated of the clay materials include bentonite, kaolinite, montmorillonite, and zeolites to remove Pb^2+^ [223]. These phyllosilicate clays have stratified structures and negatively charged surfaces, which enable the attraction as well as exchange of ions with metal cations such as Pb^2+^. Swelling capacity and interlayer spacing are particularly high in montmorillonite, which enables easy and effective absorption of Pb through surface complexation [225].

Crystalline aluminosilicates (zeolites) are three-dimensional microporous frameworks that have excellent ion-exchange capabilities and heavy metal selectivity [226]. Natural zeolites, including clinoptilolite, have been reported to have a strong affinity to Pb^2+^, attributed to their high surface negative charge density [226]. The modified or artificial zeolites tend to have better removal efficiencies and quicker kinetics than the natural ones [227].

The fibrous magnesium-rich clay minerals (palygorskite and sepiolite) have also been found to hold potential because of their tubular networks and large specific surface areas [228]. These minerals can entrap the Pb^2+^ ions within their channels, resulting in high levels of immobilization. The adsorption capacity of clays and minerals can be enhanced by a significant margin through the functionalization of the surface and the modification of the organic ligands, acids, or metal salts [229]. Examples include acid-activated bentonite with a higher surface area and porosity, and surfactant-modified clays with a higher affinity to Pb^2+^ by hydrophobic and ionic interactions [230].

The adsorption of Pb^2+^ by clay minerals usually follows Langmuir or the Freundlich isotherms, suggesting adsorption is either monolayer or multilayer, depending on the heterogeneity of the surface [231]. The solution pH (optimal is about 5–6), contact time, and temperature are also parameters that influence the adsorption process greatly [223].

Although clay-based adsorbents have positive sides, there are also some drawbacks. The composition and structure of natural minerals may influence the adsorption consistency and performance. Also, regenerating clay-based materials can be associated with the structural degradation or the decreased capacity during the repeated cycles [45]. Nonetheless, because of their sustainability, easy handling, and compatibility with other remediation methods, clay and mineral adsorbents have been major candidates in integrated Pb remediation systems. Table 6 summarizes major clay and mineral adsorbents that have been applied to remediate Pb^2+^ with an emphasis on their composition, methods of treatment, and their reported adsorption capacities.

**Table 6:** Comparative summary of clay- and natural mineral-based adsorbents for Pb^2+^ removal.

Material Type	Composition/Origin	Modification/Treatment	Pb^2+^ Adsorption Capacity (mg/g)	Key Advantages	Reference
Bentonite clay	Naturally occurring aluminosilicate clay mineral (rich in montmorillonite)	Used in natural form	>99%	Excellent removal efficiency for multiple heavy metals low-cost, natural, and eco-friendly adsorbent	[232]
Bentonite clay	Naturally occurring clay mineral	No chemical modification	0–60 mg/g	Low-cost and environmentally friendly adsorbent Effective for treating polluted water	[233]
Montmorillonite	Montmorillonite clay	Starch-modified montmorillonite	21.5 mg/g	Simple and low-cost modification process	[234]
Natural illitic clay	Collected from the Marrakech region, Morocco	Used in natural form	15.90 mg/g	Natural, low-cost, and eco-friendly adsorbent	[235]
Natural clay	mainly composed of silica (SiO_2_), alumina (Al_2_O_3_), iron oxide (Fe_2_O_3_), and magnesium oxide (MgO)	Used in natural form	86.4 mg/g (>95%)	Natural, low-cost, and eco-friendly adsorbent	[236]
Montmorillonite clay	Purified carbon-based sorbent used for medical purposes	Acid-processed to increase surface activity	5.98 mg/g (75%)	Safe and edible sorbents suitable for medical/therapeutic use	[237]
Bentonite clay	Acid-activated bentonite	Bentonite treated with acid to enhance surface area	21.36 mg/g	Low-cost and effective adsorbent for Pb^2+^ and Cu^2+^ removal Enhanced surface area and porosity	[238]
Activated bentonite–alginate	activated bentonite clay and sodium alginate	Bentonite activated with acid or thermal activation and incorporated into an alginate matrix	107.52 mg/g	Excellent reusability Stable performance in the presence of competing salts	[239]
Nanoscale zero-valent iron composite	Activated carbon as support	Synthesized NZVI/AC composite with ultralow iron loading	59.35 mg/g (95%)	Higher adsorption than AC alone	[240]
Natural zeolite and bentonite	Naturally occurring minerals: Zeolite and Bentonite	Used in natural form	moderate to low adsorption	Naturally available, low-cost, and eco-friendly. Suitable for application in both calcareous and sandy soils	[241]
Kaolinite clay	Natural kaolinite	Kaolinite system treated with Ca-silicate and Mg-silicate	>49.66%	Simultaneous carbon immobilization enhances environmental benefit	[242]
MoS_2_@Kaolin composite	Consisting of molybdenum disulfide (MoS_2_) nanosheets	Synthesized via a facile one-step hydrothermal method, forming MoS_2_ nanosheets on the kaolin surface	280.39 mg/g	Excellent regeneration and selectivity in the presence of competing ions	[243]
Montmorillonite clay	Natural montmorillonite clay	No chemical modification	~55 mg/L in solution	Demonstrates interactions between clay, microbes, and heavy metals	[244]
Montmorillonite composite	Consisting of carbon (C) and molybdenum disulfide (MoS_2_) nanosheets	one-step solvothermal method using glucose	187.0 mg/g	Electrostatic interaction, surface diffusion, and formation of PbMoO_4_ on the surface Excellent selectivity and stability for Pb^2+^ removal	[224]
Natural kaolinite	Silicate clay minerals	No chemical modification	7.75 mg/g	Naturally abundant clays and Competitive sorption capacity for multiple metals	[245]
Calcium bentonite clay	Obtained from the El Alamein region, northern Egypt	Acid and alkali treatment	13 ± 0.04 mg/g	Low-cost, abundant, and eco-friendly Egyptian clay for wastewater treatment	[246]
Natural Bentonite (NB)	Obtained from the El Alamein region, northern Egypt	Used in natural form	9 ± 0.03 mg/g	Low-cost, eco-friendly, and abundant	[246]
Na-bentonite	sodium bentonite clay combined with sawdust	composite mixture prepared with Na-bentonite	58%	Low-cost natural composite material	[247]
Kaolinite-based clay	Natural kaolinite clay	Used in natural form	69.93 mg/g (>98%)	high availability, ease of preparation, and low cost	[225]

Note: The adsorption rate is different. It depends on the initial concentration and many other parameters.

## 9. Integrated and Hybrid Approaches

Integrating complementary remedial practices such as biological-, adsorptive-, and phytotechnologies, biochar-based adsorption, and nanomaterial improvements, advances secure greater Pb removal efficiencies of complex wastewater at reasonable costs, without secondary pollution and operational limitations. Hybrid approaches utilize quick physicochemical capture (precipitation, adsorption, magnetic separation) with slower yet sustainable biological (bio-sorption, bioaccumulation, phytoremediation, microbial transformation) processes. This is why integrated and hybrid systems are undergoing development to enhance the efficiency of treatment, cost-efficiency, and environmental sustainability [248].

Nanoscale modification of biochar (nanobiochar) significantly enhances surface area, active binding sites, and functionalization in a tailored form, e.g., oxygenated functional groups or metal-chelating ligands [249]. Microbe-nanotech hybrid interaction may enhance microbial activities by focusing the contaminants around the biomass, providing reactive surfaces for the transfer of electrons, or by magnetic harvesting of biomass loaded [250]. Some of these strategies are the immobilization of metal-adsorbing microbes on nanoparticle functionalized supports, and biosorption in conjunction with magnetite nanoparticles improves microbial stress tolerance. Such strategies provide quicker kinetics, larger capacity, and simplified separation, though there should be a thorough consideration of nanoparticle toxicity [250].

The use of phyto-biochar and plant-microbe consortia led to better rhizosphere, activation of beneficial microbes, and immobilization of Pb around roots, which are better for promoting plant survival and uptake in phytoextraction [251]. The combination of tolerant plant species with biochar and plant growth-promoting rhizobacteria or fungi raises biomass, enhances soil health, and increases Pb uptake.

The integration of a physicochemical method, e.g., coagulation/flocculation, adsorption, precipitation, and a biological polishing step like biosorption bed, constructed wetlands, or algal systems, influences the advantages of both systems. The quick elimination of Pb by physicochemical methods helps to reduce toxicity shocks to biological systems, whereas living systems offer long-term attenuation and ecological restoration [248]. While nano-biochar and microbe-nanotech hybrids are promising, they are still affected by challenges on stability over a long period, ecotoxicity of nanoparticles, regeneration protocols, and scalability [252]. The pilot tests should focus on the real-effluent demonstrations, lifecycle analysis, and addition of Pb recovery to improve sustainability.

Although much progress has been made in the research of integrated and hybrid methods of remediation of Pb, they are still subject to many limitations that make it difficult to widely use them in practice. The obstacles are of technical, environmental, and economic nature, and are even more complex in a large-scale implementation and regulatory control [248]. The majority of integrated systems have been tested in small pilot or controlled laboratory environments. Biological stability, microbial consortia, and phytoremediation systems are susceptible to changes in pollutants, seasonal changes, and pathogen competition [253]. Bridging these gaps requires combined policies in which the wastewater treatment performance is related to the life cycle assessment of the remediation material applied, and standardized testing protocols for the hybrid systems should be formulated.

## 10. Comparative Analysis and Performance Evaluation

The effectiveness of Pb^2+^ remediation technologies is determined by a variety of factors, such as removal efficiency, adsorption capacity, cost-effectiveness, environmental sustainability, regeneration potential, and scalability [254]. The critical analysis of the variations in the parameters, which is based on comparison, will help to choose the most appropriate approach for certain site conditions, economic limitations, and regulatory provisions. Although all of these methods have their own unique strengths, they also possess certain limitations in the conditions of operation, regeneration ability, capacity to remove, and financial viability. Thus, the interest in the investigation of integrated and hybrid remediation systems that integrate the advantages of various methods to improve the performance and address the limitations of single technologies is increasing. Examples include microbial-nanocomposite systems, biochar-nanoparticle composites, and plant-microbe associations, which are gaining momentum as an attractive exploitative approach, utilizing biological activity, high sorption capacity, and functional stability [255,256]. However, a collective insight into the comparative output, sustainability, and realistic application of these varied technologies is still lacking. Table 7 summarizes a comparative assessment of primary Pb^2+^ remediation technologies, their performance parameters, cost effectiveness, scalability, and practical limitations of these technologies as reported in the literature.

## 11. Future Perspectives

The removal of Pb pollution has achieved significant achievements using microbial, plant-based, adsorptive, and nanotechnological methods. Nevertheless, there are a number of restrictions and issues with scalability, cost-effectiveness, selectivity, environmental compatibility, and regulation standardization. The improvement of sustainable and circular Pb remediation systems requires a multidisciplinary roadmap addressing innovation of effective materials, integration of digital devices, practical testing, and policy alignment.

The development of sustainable, low-cost, and environmentally friendly materials in the arrangement for Pb remediation is a significant direction in the future known as Sustainable Material Development. Agricultural wastes, including rice husk ash, banana peels, coconut shells, microbial biomass, and nanomaterials produced by the green process, also have great potential for scalable biosorbents and adsorbents. Such materials should be highly selective, have fast kinetics, and be stable in complicated wastewater systems. Biochar-based composite, hybrid hydrogel, and bio-inspired nanostructure development can offer improved surface area and functionalization of Pb binding. Invest in green synthesis, recyclable, and bio-based adsorbents derived from waste materials.

The application of machine learning (ML), artificial intelligence (AI), and computational modeling is a more recent and rapidly growing research area of Pb remediation. These tools can be used to maximize the experimental parameters, forecast adsorption behavior, and model large-scale treatment. Develop and validate AI-based predictive models of optimization and adaptation of the system to real environmental conditions. The use of artificial neural networks (ANNs), genetic algorithms (GAs), and support vector machines (SVMs) in modeling sorption kinetics, predicting Pb removal efficiency, and determining process robustness under changing environmental conditions has been conducted recently.

There is an urgent policy integration and circular economy requirement to incorporate Pb remediation into the overall policies of the circular economy and zero-waste. This involves not only the elimination of Pb in the polluted areas but also reclaiming and reusing the bound metal by the resource recovery techniques like electrochemical regeneration, solvent extraction processes, or bioleaching.

Regulatory frameworks should be redesigned to promote compulsory cleanup of old Pb locations, subsidies on the adoption of green technologies, the standardization of remediation performance indicators, and the alignment of environmental protection, community health, and industrial solid waste management across multiple sectors. Regulatory frameworks should encourage the circular economy of Pb remediation by recovery, reuse, and waste management.

Additionally, national and international policy discussions should include community involvement, environmental justice, and international equity in Pb cleanup. The future strategies should be in line with the United Nations Sustainable Development Goals (SDGs), which are clean water and sanitation, as well as responsible consumption and production.

## 12. Conclusions

This critical review indicates that microbial, plant-based (phytoremediation), adsorptive, and nanotechnology-based remediation strategies offer different mechanistic routes by which Pb^2+^ can be immobilized and removed. However, a combination of these complementary approaches is a more sustainable, high-efficiency, and scalable means of removing lead-contaminated wastewater. All the approaches have specific strengths and weaknesses on the aspects of removal effectiveness, cost-effectiveness, scalability, environmental friendliness, and regeneration capability.

Microbial approaches have displayed an outstanding promise of Pb biosorption and bioaccumulation, especially when operating in optimal environments. Phytoremediation offers a relatively eco-friendly, aesthetically friendly method of large-scale remediation, particularly in low-contamination sites, but suffers due to low kinetics and plant stress. Biosorbent, activated carbon, and agro-waste-based adsorption methods have been extensively studied as they are relatively simple and effective. Nanotechnology-based interventions, particularly the use of functionalized nanoparticles, nanocomposites, have better surface properties, fast kinetics, and regeneration at the expense of toxicity and high cost of synthesis.

An integrated remediation framework combines the strengths of each approach and reduces the limitations of the others for the best potential of Pb remediation. Moreover, to achieve scalable and durable Pb remediation solutions, sustained material innovation, artificial intelligence optimization, practical, and policy-level contributions are necessary. Finally, green engineering, digital technology, and policy reform are all expected to converge so that Pb removal technologies are not only effective but also sustainable and equitable.

## Figures and Tables

**Table 1:** Major microorganisms employed in the removal of Pb in wastewater, their type, their removal mechanism, adsorption/removal efficiency, and source.

Microorganism	Type	Mechanism	Adsorption/Removal Efficiency (%)	Reference
*Bacillus amyloliquefaciens*	Bacteria	Biosorption	10,000 ppm	[74]
*Oceanobacillus profundus* KBZ 3-2	Bacteria	Biosorption	97%	[78]
*Bacillus* sp. *AKVPbR02*	Bacteria	Bioflocculation	74–89.5%	[79]
*Bacillus cereus*	Bacteria	Bioaccumulation	1000 mg/L	[80]
*Pseudomonas stutzeri* and *Cupriavidusmetallidurans*	Bacteria	Bioaugmentation	71.02%	[81]
*Bacillus subtilis X3*	Bacteria	Bioadsorption	590.49 mg/g	[51]
*Serratia marcescens*	Bacteria	Biosorption; bioprecipitation	97.57%	[82]
*Chlorella* sp. *MOW 12*	Algae	Surface adsorption, intracellular uptake	86–93%	[83]
*Anabaena* sp.	Cyanobacteria	Chelation, bioaccumulation	98.90%	[50]
*Nostoc muscorum*	Cyanobacteria	Chelation, bioaccumulation	88%	[50]
*Saccharomyces cerevisiae*	Yeast	Surface adsorption	12 mg/g	[60]
*Synechocystis* sp. PCC6803	Algae	Adsorption	62.63 mg/g (88.89%)	[84]
*Tolypthrix ceytonica*	Cyanobacteria	Bioaccumulation	94.22%	[49]
*Anabaena variabilis*	Cyanobacteria	Bioaccumulation	98.61%	[49]
*Penium margaritaceum*	Algae	Adsorption	3.4 mg/g (55.4%)	[85]
*Spirulina*	Cyanobacterium	Entrapment	282.17 mg/g	[86]
green algae	Algae	Adsorption	71–75%	[87]
*Undaria pinnatifida*	Marine Algae	Biosorption	32.6 ppm (67.4%)	[88]
*Gracilaria changii*	Red Algae	Adsorption	0.1 mg/g.	[89]
*Hizikia fusiformis*	Algae	Adsorption	167.73 mg/g	[90]
*Microcystis aeruginosa*	Cyanobacterium	Adsorption	81.3 mg/g (90%)	[91]

Notes: Removal efficiencies may vary depending on contact time, initial Pb concentration, and biomass dosage. Mechanisms are often a combination of surface adsorption, ion exchange, and metabolic uptake.

**Table 2:** Major agro-waste-based adsorbents and fruit peel-based adsorbents with their treatment or modification techniques, reported Pb adsorption capacity or removal percentage, optimum operating conditions, and references.

Adsorbent	Treatment/Modification	Adsorption Capacity (mg/g)/Removal%	Optimal Conditions	Reference
Orange peel cellulose	Chemically treated	98.33%	pH 7, contact time 12 h, temperature 28 °C,	[100]
Pineapple waste	Chemically treated with NaOH	85.88%	pH 2–4, contact time 60 min, temperature 28 °C,	[121]
Grape peel	Raw grape fruit peel powder	57.9 ± 0.9 mg/L	pH 4, contact time 60 min, temperature 50 °C	[122]
Apple peel	Chemically treated and breaded	73%	pH 7, contact time 6 h, temperature 25 °C	[95]
Lemon peel	Raw lemon peel powder	99%	pH 10, contact time 24 h, temperature 20 °C	[123]
Mango peel	Raw mango peel powder	96%	pH 4, contact time 24 h, temperature 20 °C	[123]
Banana peel	Raw banana peel powder	98%	pH 10, contact time 24 h, temperature 20 °C	[123]
Orange peel	Raw orange peel powder	98%	pH 10, contact time 24 h, temperature 20 °C	[123]
Watermelon rind	Raw watermelon rind powder	230.5 mg/g	pH 5, contact time 24 h, temperature 25 °C	[124]
Lemon peel	Powdered and beaded	5.67 mg/g 86%	pH 5–6, Contact time 90 min	[99]
Potato peel	Raw potato peel	256.17 ± 2.17 mg kg^−1^	pH 4–6, contact time 60 min, temperature 22 °C	[125]
Passion peels	Raw passion peel powder	1077.47 ± 12.56 mg kg^−1^	pH 4–6, contact time 60 min, temperature 22 °C	[125]
Orange peel	Raw orange peel powder	264.55 ± 1.46 mg kg^−1^	pH 4–6, contact time 60 min, temperature 22 °C	[125]
Orange peel	Modified with NaOH and CaCl_2_	209.8 mg/g	pH 5.5, contact time 120 min, temperature 25 °C	[126]
Orange peel	Raw orange peel powder	19.146 mg/g (95.73%)	pH 2, contact time 40–60 min, temperature 50 °C	[127]
Lemon	Raw lemon peel powder	19.318 mg/g (96.59%)	pH 2, contact time 40–60 min, temperature 50 °C	[127]
Banana	Raw banana peel powder	19.180 mg/g (95.89%)	pH 4, contact time 40–60 min, temperature 50 °C	[127]
Watermelon	Raw watermelon peel powder	19.392 mg/g (96.96%)	pH 2, contact time 40–60 min, temperature 50 °C	[127]
Potato peel	Raw potato peel	217 mg/g	pH 6, contact time 24 h, temperature 50 °C	[128]
Pomegranate Peel	Raw pomegranate peel powder	335 mg/L	pH 5.5, Temperature 30 °C, Contact time 120 min	[129]
Corn silk	Not modified	90 mg/g	pH 5.0, Temperature 293–313 K, Contact time 60–120 min	[130]
Rice husk	Chemically modified with PTFE	98.38%	pH 7.0, initial concentration of lead (10, 55, and 100 μg/L), Contact time ~30 min	[102]
Wheat husk	Modified via phosphoric acid	~72.2%	pH 5.5, Room temperature, Contact time 6 h	[104]
Sawdust	Treated with H_2_SO_4_ and NaOH	91.30%	pH 5, Temperature ≈ 23 °C, Contact time ~40 min	[105]

Notes: Adsorption capacities vary depending on adsorbent preparation, experimental setup, and Pb concentration. The optimal pH usually ranges between 5 and 6 due to the speciation of Pb and the adsorbent surface charge. Contact times range from 30 min to 2 h, depending on the adsorbent and Pb concentration.

**Table 7:** Comparative summary of Pb remediation of different methods.

Technique	Adsorption Capacity (mg/g)	Removal Efficiency	Cost-Effectiveness	Environmental Sustainability	Regeneration Potential	Scalability	Key Challenges	Reference
Microbial approaches	20–590 mg/g	Moderate to high	Moderate	High	Moderate	Moderate	Slow kinetics, environmental sensitivity	[51,84]
Agro-waste/Fruit peels	30–1077 mg/g	Moderate to high	High	Very high	Low to moderate	Moderate	Fouling, limited selectivity	[125,129]
Biochar	45–1429 mg/g	High	High	High	High	High	Inconsistent Pb adsorption capacity	[166]
Activated carbon	100–591 mg/g	Very high	Moderate to low	Moderate	High	High	High cost and limited regeneration efficiency	[171]
Phytoremediation	20–9284 mg/kg DW	Low to moderate	High	Very high	Low (biomass disposal)	Moderate to high	Long duration, affected by the climate	[145,151]
Nanotechnology-based materials	20–673 mg/g	Very high	Low to moderate	Low to moderate	High	Low to moderate	Toxicity concerns, high production cost	[208]
Clay/Natural minerals	7–280 mg/g	High	Very high	High	Moderate to high	High	Low adsorption capacity and slow kinetics for Pb removal	[243,245]
Integrated hybrid systems		Very high	Variable	High	High	High	Complexity in design and maintenance	[248]

## Data Availability

No new data were created or analyzed in this study. Data sharing is not applicable to this article.
